# Quantitative sensory testing and norepinephrine levels in REM sleep behaviour disorder – a clue to early peripheral autonomic and sensory dysfunction?

**DOI:** 10.1007/s00415-021-10675-7

**Published:** 2021-06-25

**Authors:** Julia Koch, Kira Willemsen, Imis Dogan, Roman Rolke, Jörg B. Schulz, Johannes Schiefer, Kathrin Reetz, Andrea Maier

**Affiliations:** 1 Department of Neurology, Medical Faculty RWTH Aachen University, Pauwelsstraße 30, D-52074 Aachen, Germany; 2 JARA-BRAIN Institute Molecular Neuroscience and Neuroimaging, Forschungszentrum Jülich GmbH, Medical Faculty RWTH Aachen University, Pauwelsstraße 30, D-52074 Aachen, Germany; 3 Department of Palliative Medicine, Medical Faculty RWTH Aachen University, Pauwelsstraße 30, D-52074 Aachen, Germany

**Keywords:** Idiopathic REM sleep behaviour disorder, Autonomic dysfunction, Quantitative sensory testing, Norepinephrine levels, Somatosensory dysfunction

## Abstract

**Introduction:**

Studies have reported autonomic impairment in patients with idiopathic REM sleep behaviour disorder (iRBD), which is considered a prodromal stage of alpha-synucleinopathies. It is still debated whether central or peripheral pathologies are first manifestations of alpha-synucleinopathies. This study aimed to characterize autonomic and somatosensory function in iRBD patients.

**Methods:**

This cross-sectional prospective case–control study included 17 iRBD patients (mean age 66.3 ± 9.2 years) and 16 healthy controls (HCs, 66.6 ± 11.3 years). Quantitative sensory testing, neurological and neuropsychological assessments, norepinephrine blood plasma levels, tilt table examination with orthostatic blood pressure, and heart rate variability were carried out. Longitudinal data of 10 iRBD patients, including neurological, neuropsychological, and tilt table examination, were assessed.

**Results:**

iRBD patients more frequently presented with orthostatic dysfunction than HCs (70.6% vs. 6.3%, *p* < 0.0001). Supine norepinephrine plasma levels were normal, but lower in iRBD (249.59 ± 99.78 pg/ml iRBD, 354.13 ± 116.38 pg/ml HCs, *p* < 0.05). Quantitative sensory testing revealed impaired cold (CDT) and vibration detection thresholds (VDT) on the foot in iRBD (CDT foot iRBD − 1.24 ± 0.31, HCs − 9.89E-17 ± 0.25, VDT iRBD − 1.11 ± 0.47, HCs − 1.46E-16 ± 0.25, *p* < 0.05). Cold detection thresholds differed between the foot and hand among iRBD patients (foot − 1.24 ± 0.31, hand − 0.56 ± 0.25, *p* < 0.05). Longitudinal data revealed an increase in maximum systolic and diastolic orthostatic blood pressure changes and a decrease in the Valsalva ratio in the follow-up group (*p* < 0.05).

**Conclusion:**

This study revealed autonomic dysfunction with somatosensory impairment, and decreased norepinephrine levels in iRBD, which may serve as a possible prodromal marker for developing alpha-synucleinopathy.

**Supplementary Information:**

The online version contains supplementary material available at 10.1007/s00415-021-10675-7.

## Introduction

Idiopathic REM sleep behaviour disorder (iRBD) is characterized by pathological dream-enacting behaviour during REM sleep and is considered a premotor stage of alpha-synucleinopathies (α-SYN), such as Parkinson’s disease (PD) or multiple system atrophy (MSA) [1, 2]. It is already known that several autonomic symptoms can occur in this prodromal stage of α-SYN [3, 4]. We recently described the prevalence of transient orthostatic hypotension (OH) in iRBD [5]. In addition to obstipation and olfactory dysfunction, OH is one of the most common autonomic features of α-SYN [6]. Regarding the onset and manifestation of PD in iRBD, there remains debate whether central or peripheral pathology is the first to appear. One hypothesis posits the spread of alpha-synuclein towards the brain from the peripheral nervous system to the central nervous system [8]. Recent studies have demonstrated the presence of various peripheral autonomic impairments and of alpha-synuclein aggregates in the skin; first metabolic brain changes; and a reduction in intraepithelial nerve fibre density in iRBD [5, 9–12]. Here, we characterized peripheral autonomic and somatosensory functions in iRBD by means of noninvasive quantitative sensory testing (QST), norepinephrine (NE) blood plasma levels, orthostatic BP, and heart rate changes.

## Materials and methods

### Subjects

Seventeen male iRBD patients from the prospective natural history cohort study of the German RBD Study Group were examined at the Department of Neurology, RWTH Aachen University, between February 2016 and May 2018 and were compared to 16 healthy controls (HCs) matched for sex and age. iRBD was screened using the RBD Screening Questionnaire and was polysomnographically confirmed by diagnostic criteria established by the American Academy of Sleep Medicine, including abnormal behaviour during REM sleep, electromyographic activity, and dream enactment, as described previously in Schrempf et al. [12]. The study was approved by the institutional ethics review board at RWTH Aachen University (EK 231/09) and carried out under the terms of the Declaration of Helsinki. All participants gave written informed consent prior to study enrolment. Exclusion criteria were:

1. clinical motor signs, as assessed through detailed neurological examination and the Unified Parkinson’s Disease Rating Scale (UPRDS-III) [13] (cut off > 5);
2. sleep apnoea syndrome (higher than grade two);
3. severe heart failure;
4. long-standing diabetes mellitus with HbA1c > 7% as well as deficits of vitamin B12 or folic acid; in HC subjects, signs suggestive for peripheral neuropathy including abnormalities in nerve conduction studies and reduced clinical vibration detection.

In addition to carrying out a cross-sectional, case–control design, we also analyzed longitudinal data in a subgroup of ten iRBD patients with follow-up assessments following their inclusion in the natural history study between 2010 and 2016.

### Autonomic function and norepinephrine plasma levels

The autonomic testing battery included sympathoneural function (BP changes during head up-tilt, NE blood plasma levels, taken both supine and standing) and cardiovagal function (supine heart rate changes, during deep breathing, orthostasis as well as the Valsalva manoeuvre). Tests were always performed in a fasted, standardized manner in the morning without morning medication, smoking or caffeine intake for at least 12 h. BP, breathing rate, and heart rate were recorded continuously using a Finometer Midi (Finapres Medical Systems B.V., Enschede; Netherlands) and Fan 4.1.0 (BioSign GmbH, Ottenhofen, Germany). BP measurement was consistently assessed in a standardized manner, using calibration between the brachial arterial pressure and the finger artery pressure, correcting for height, and continuous recording at the cardiac level. During two minutes of deep breathing, defined as six inspirations per minute, respiratory sinus arrythmia (RSA) was quantified as the median difference between the maximal and minimal interbeat (R–R) intervals per respiratory cycle (diffR-Rmax-R-Rmin). Parameters of heart rate variability (i.e., coefficient of variation during rest, Ewing coefficient in upright position, and Valsalva ratio), were calculated and rated pathologically, as previously described [5]. The tilt table examination was performed supine for 10 min, followed by 20 min in a 70° upright position. OH in this study included all variants of OH and were defined as follows: classic OH, if systolic (> 20 mmHg) or diastolic (> 10 mmHg) BP dropped within three minutes of standing compared to mean supine BP values before [7]; transient OH, if BP dropped transiently and stabilized within five minutes after standing; and delayed OH, if the abovementioned BP dropped beyond three minutes of standing [7]. The maximum heart rate rise and the systolic/diastolic BP decrease compared to baseline, were calculated. NE plasma levels were measured in supine blood samples taken after ten minutes of rest (NE supine) and after ten minutes in an upright position (NE upright). In healthy persons, plasma levels of NE double within five minutes after postural change and stabilize BP [14]. Neurogenic orthostatic hypotension (NOH) is characterized by reduced NE plasma levels and/or a reduced noradrenergic responsiveness to postural change [14].

### Quantitative sensory testing

According to the protocol of the German Research Network on Neuropathic Pain (DFNS), QST was consistently performed in the same procedure on the left hand dorsum and left foot dorsum, as described previously [15]. Standardized instructions were used to quantify the functional state of the somatosensory system, including nerve fibre function of small fibres; C- and Aδ-fibres, and large fibres; Aβ-fibres, as well as their pathways to the brain. QST included all thermal and mechanical detection and pain thresholds necessary for assessing a complete sensory phenotype.

### Neuropsychological and neurological assessment

Neuropsychological and neurological assessments were performed, as described previously [12]: the Montreal Cognitive Assessment (MoCA), Unified Parkinson’s Disease Rating Scale (UPDRS), and Hoehn and Yahr (HY) were administered. Typical non-motor and autonomic symptoms, as analogously described in PD, were rated by the non-motor symptoms questionnaire (NMS Quest) for comparability. Screening for depressive symptoms was performed using the Beck Depression Inventory, version II (BDI-II). Furthermore, olfactory deficits were assessed via the “Sniffin’ Sticks” identification test. Signs of polyneuropathy were ruled out by motor and sensory nerve conduction studies and vibration detection.

### Longitudinal assessment

For this autonomic subproject, we added QST and NE levels in 2016 to our natural history study (started on 09/2010). A subgroup of patients participated in follow-up assessments, allowing for longitudinal analysis of tilt table examination and neuropsychological and neurological assessments.

### Statistical analysis

We compared the differences between groups (iRBD vs. HCs) using the two-sample *t* test and Mann–Whitney *U* test, as appropriate. Fisher’s exact test was used to analyse NMS Quest frequency distributions and heart rate variability parameters. Smelling and heart rate variability parameters were compared between groups using ANCOVA with age as the covariate. Paired supine and upright systolic and diastolic BP, heart rate, NE plasma levels, and QST data were analysed using ANOVA with repeated measures. Cohen’s *f* was used as the effect size, defined as *f* = 0.10 mild, *f* = 0.25 moderate and *f* = 0.40 for severe effects. For QST data, ANOVA was followed by Fisher’s LSD post hoc tests. Most QST values were normally distributed in log-space and transformed logarithmically before calculating z-scores in relation to log-data of HC subjects. For correlation analyses, we used Spearman’s coefficient. To compare follow-up and baseline assessments in the subgroup of iRBD, the paired sample *t* tests and Wilcoxon signed-rank tests were used. A *p* value of ≤ 0.05 was considered significant. Statistical analysis was performed with IBM SPSS Statistics Version 25 (IBM SPSS Statistics for Mac, Version 25.0) and Statistica software for Windows 7.1 (StatSoft Inc., USA).

## Results

### Sample characteristics

Seventeen male iRBD patients (mean age 66.3 ± 9.2 years, mean disease duration since diagnosis 5.9 ± 3.5 years) were compared with 16 male HCs (mean age 66.5 ± 11.3 years). See Table 1 for detailed sample characteristics and missing data. In nine iRBD patients, cranial MRI scans were able to be performed without any signs of structural abnormalities (e.g., atrophy referring to MSA). Four iRBD patients (23.53%) showed electroneurographical signs of sensorimotor polyneuropathy without clinical symptoms and without a significantly impaired vibration detection threshold, as compared to iRBD patients with physiological nerve conduction studies. Subjects were diagnosed with following comorbidities: obesity (12.50% iRBD, 26.70% HCs), nephropathy (5.89% iRBD), arterial hypertension (17.65% iRBD, 43.75% HCs), heart disease (29.41% iRBD, 6.25% HC), early type II of diabetes mellitus (6.25% HC), and smoking (29.41% iRBD, 12.50% HCs). In terms of medications, two iRBD patients (11.76%) were taking a serotonin–norepinephrine reuptake inhibitor (duloxetine), three iRBD patients (17.65%) and seven HCs (43.75%) were taking antihypertensive medication, two iRBD patients (11.76%) and one HC (6.25%) used to take beta-1-specific adrenergic antagonists, and two HCs (12.50%) used local eye drops containing nonspecific beta-adrenergic antagonists for glaucoma. Hyposmia, evaluated via the Sniffin’ Sticks identification test, was more frequent in iRBD 52.94% vs. in HCs 0% (median olfaction score, right: 6 (interquartile range 5–9) in iRBD vs. 13.5 (interquartile range 12–14.5) in HCs, *F* = 23.17 *p* < 0.0001; left: 9 (interquartile range 6–10) in iRBD vs. 14 (interquartile range 11.5–15) in HCs, *F* = 12.26 *p* = 0.004), and iRBD patients displayed more depressive symptoms (BDI-II 7.77 ± 8.87 iRBD vs. 1.5 ± 2.42 HCs, *U* =  − 2.56, *p* = 0.014). The incidence of motor and non-motor symptoms was also significantly higher in iRBD patients than in HCs, as expected (Table 1).

**Table 1:** Demographic and clinical data of patients with idiopathic REM sleep behaviour disorder compared to healthy controls (cross-sectional) and longitudinal assessments of patients with idiopathic REM sleep behaviour disorder baseline vs. follow-up assessments

	Cross-sectional group			Longitudinal assessment iRBD			
	iRBD	HC	*p* value	baseline	follow-up		*p* value
Demographics							
Subjects, *n*	17	16	n.s	10	10		n.s
Sex	17 m, 0 f	16 m, 0 f	n.s	10 m, 0 f	10 m, 0 f		n.s
Age, years	66.25 ± 9.17	66.54 ± 11.33	n.s	**61.54 ± 11.97**	**65.27 ± 11.08**		** < 0.0001**
Duration of iRBD, years	5.85 ± 3.47	n.a	n.s	2.31 ± 2.25	5.95 ± 3.08		**0.001**
RBD Quest	8.53 ± 2.76**	0.75 ± 1.39	** < 0.0001**	9.90 ± 3.00	8.70 ± 2.71*		**0.024**
UPDRS III	2.29 ± 1.90**	0.19 ± 0.54	** < 0.0001**	0.90 ± 1.29	1.90 ± 2.23		n.s
Sympathoneural							
NE supine, pg/ml	249.59 ± 99.78*	354.13 ± 116.38	**0.017**	n.a	n.a		n.a
NE upright, pg/ml	579.12 ± 235.41	760.63 ± 303.45	n.s	n.a	n.a		n.a
Mean supine systolic BP, mm Hg	125.45 ± 20.92	124.74 ± 20.28	n.s	117.04 ± 11.43	125.87 ± 21.22		n.s
		*n* = 15					
Mean upright systolic BP, mm Hg	123.17 ± 21.62	128.72 ± 29.74	n.s	120.31 ± 12.68	121.21 ± 18.62		n.s
		*n* = 15					
Mean supine diastolic BP, mm Hg	68.41 ± 9.84	69.72 ± 12.12	n.s	65.89 ± 7.16	70.10 ± 8.52		n.s
		*n* = 15					
Mean upright diastolic BP, mm Hg	71.82 ± 12.59	75.66 ± 14.61	n.s	73.20 ± 7.63	73.10 ± 12.61		n.s
		*n* = 15					
Mean HR supine, beats/min	60.85 ± 9.14	58.75 ± 8.33	n.s	64.32 ± 9.6	62.55 ± 9.94		n.s
		*n* = 14		*n* = 8			
Mean HR upright, beats/min	68.71 ± 10.88	64.29 ± 6.70	n.s	**72.98 ± 10.60**	70.61 ± 11.71*		**0.019**
		*n* = 14		*n* = 8			
Systolic BP change 3 min standing, mm Hg	− 0.96 ± 9.00	2.13 ± 6.31	n.s	− 1.21 ± 4.89	− 0.25 ± 10.15		n.s
		*n* = 15					
Diastolic BP change 3 min standing, mm Hg	3.32 ± 13.26	7.70 ± 6.83	n.s	6.90 ± 9.05	3.52 ± 16.97		n.s
		*n* = 15					
HR change 3 min standing, beats/min	7.48 ± 8.26	6.20 ± 7.15	n.s	10.43 ± 8.27	9.34 ± 7.56		n.s
		*n* = 14					
Max systolic BP change, mm Hg	− 41.45 ± 16.88*	− 23.67 ± 16.23	**0.005**	**-24.50 ± 11.70**	− 42.27 ± 20.01*		**0.024**
		*n* = 15					
Max diastolic BP change, mm Hg	− 21.35 ± 11.48*	–10.92 ± 8.76	**0.008**	− **12.79 ± 7.99**	− 21.60 ± 13.34*		**0.037**
		*n* = 15					
Max HR raise, beats/min	20.32 ± 7.99	18.39 ± 13.16	n.s	20.87 ± 8.4	21.82 ± 9.33		n.s
		*n* = 14		*n* = 8			
Max BP change mean systolic, mm Hg	− 2.28 ± 15.98	3.98 ± 14.28	n.s	3.27 ± 14.47	− 4.66 ± 19.53		n.s
		*n* = 15					
Max BP change mean diastolic, mm Hg	3.41 ± 11.04	5.94 ± 7.30	n.s	7.31 ± 7.85	3.0 ± 13.99		n.s
		*n* = 15					
Cardiovagal							
RSA difference BPmax/BPmin, ms	102.25 ± 71.29	124.13 ± 60.47	n.s	81.11 ± 80.62	99 ± 69.43		n.s
	*n* = 16	*n* = 15		*n* = 9			
Heart rate variability supine, ms	4.02 ± 2.32	6.87 ± 6.89	n.s	4.21 ± 1.72	3.83 ± 2.21		n.s
		*n* = 15		*n* = 8			
Valsalva ratio (BPmax/BPmin)	1.30 ± 0.12	1.44 ± 0.43	n.s	1.46 ± 0.27	1.29 ± 0.11*		**0.007**
	*n* = 16	*n* = 12		*n* = 9	*n* = 9		
Ewing 30:15 quotient	1.36 ± 0.77	1.16 ± 0.11	n.s	1.15 ± 0.11	1.12 ± 0.07		n.s
		*n* = 14		*n* = 9			
Non-motor symptoms							
NMS Quest	6.82 ± 3.73**	2.19 ± 2.07	** < 0.0001**	7.10 ± 4.07	6.60 ± 3.13		n.s
OH	**12 yes, 5 no**	1 yes, 15 no	** < 0.0001**	3 yes, 7 no	8 yes, 2 no*		**0.015**
BDI-II	7.77 ± 8.87*	1.50 ± 2.42	**0.014**	7.00 ± 9.24	5.70 ± 6.99		n.s
MoCA	27.41 ± 2.06	28.69 ± 1.25	n.s	27.44 ± 2.01	27.10 ± 2.28		n.s
				*n* = 9			
Olfaction left	9 (6–10)°**	14 (11.5–15)°	**0.004**	10 (7–10)°	9 (6–10)°		n.s
				*n* = 9			
Olfaction right	6 (5–9)°**	13.5 (12–14.25)°	** < 0.0001**	8.5 (7.75–10.25)°	7 (6–9.75)°		n.s
				*n* = 8			

Bold values represent the significant data

Beck Depression Inventory II (*BDI-II*), blood pressure (*BP*), female (*f*), healthy control (*HC*), heart rate (*HR*), idiopathic REM sleep behaviour disorder (*iRBD*), male (*m*), Montreal Cognitive Assessment (*MoCA*), Non-motor Symptoms Questionnaire (*NMS Quest*), norepinephrine (*NE*), not significant (*n.s.*), orthostatic hypotension (*OH*), RBD Questionnaire (*RBD Quest*), respiratory sinus arrhythmia (*RSA*), Unified Parkinson’s Disease Rating scale, part III (*UPDRS III*)

Significant differences between groups or baseline vs. follow-up at *p < 0.05, **p < 0.001, data presented as the mean ± SD, but as median (IQR) for olfaction

### Cross-sectional autonomic data

**Fig. 1:**
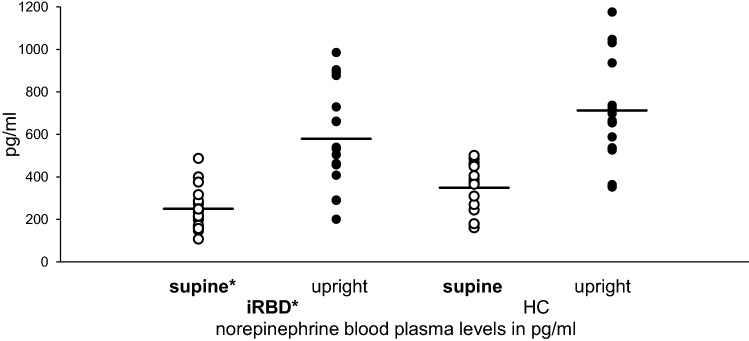
Comparison of norepinephrine blood plasma levels, supine vs. upright, in idiopathic REM sleep behaviour disorder patients compared to healthy controls. Significant difference of norepinephrine blood plasma levels in iRBD patients compared to that in HCs with significantly lower norepinephrine supine levels in iRBD patients with sustained response to postural change (NE ratio not significantly impaired) healthy controls (HCs), idiopathic REM sleep behaviour disorder (iRBD), — mean, *p < 0.05

In total, 70.59% of iRBD patients and 6.25% of HCs presented with any type of OH. Four iRBD patients had classic OH; five had transient OH; two had delayed OH; and one patient had a transient BP drop, first with stabilization and then with delayed OH. One HC tilt table examination was excluded from the analysis due to measurement errors. In addition, the following had to be excluded: one iRBD RSA Score; one iRBD Valsalva test; and four HCs who were not allowed to perform the Valsalva manoeuvre due to contraindications. In one HC, the heart rate parameters were not evaluable due to cardiac arrhythmia. BP dropped more severely in iRBD (max. change − 41.45 ± 16.88 mmHg systolic, max. change − 21.35 ± 11.48 mmHg diastolic) than in HCs (max. change − 23.67 ± 16.23 mmHg systolic, *t* =  − 3.03 *p* = 0.005, max. change − 10.92 ± 8.76 mmHg diastolic, *t* =  − 2.86 *p* = 0.008). ANOVA with repeated measures confirmed a significant difference in NE levels in iRBD vs. HCs (*F* = 5.83, *p* = 0.022, effect size *f* = 0.43) and in the supine vs. upright position, as expected (*F* = 82.64, *p* < 0.0001, effect size *f* = 1.63). With regard to individual values, NE supine was lower in iRBD (249.59 ± 99.78 pg/ml iRBD vs. 354.13 ± 116.38 pg/ml HCs, *U* =  − 2.38, *p* = 0.017), whereas the NE ratio (NE supine vs. upright) did not differ significantly between iRBD patients and HCs (Fig. 1).

**Fig. 2:**
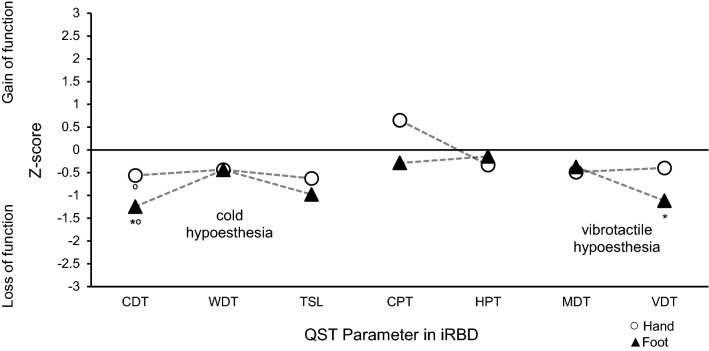
QST in idiopathic REM sleep behaviour disorder patients. Quantitative sensory testing reveals increased cold detection and vibration detection thresholds (i.e., loss of function) on the foot in iRBD patients compared to those in HCs. Cold detection threshold in iRBD patients presents length-dependent loss of function on the foot vs. on the hand *n* = 17, all parameters, except for thermal testing *n* = 16, on the left hand dorsum and left foot dorsum, cold detection threshold (*CDT*), cold pain threshold (*CPT*), healthy controls (*HCs*), heat pain threshold (*HPT*), idiopathic REM sleep behaviour disorder (*iRBD*), mechanical detection threshold (*MDT*), quantitative sensory testing (*QST*), thermal sensory limen (*TSL*), vibration detection threshold (*VDT*), warm detection threshold (*WDT*) *p < 0.05 iRBD vs. HCs, *p* < 0.05 Hand vs. Foot (ANOVA, Fisher’s LSD post hoc test), data presented as z-scores

In QST, the cold detection threshold on the left foot dorsum in iRBD patients was significantly higher than that in HCs (iRBD − 1.24 ± 0.31, HCs − 9.89E-17 ± 0.25, *p* = 0.007). In addition, the cold detection threshold on the left foot was significantly increased in comparison to the cold detection threshold on the left hand within iRBD patients (foot − 1.24 ± 0.31, hand − 0.56 ± 0.25, *p* = 0.038). The vibration detection threshold was significantly increased in iRBD patients on the foot compared to that in HCs (iRBD − 1.11 ± 0.47, HCs − 1.46E-16 ± 0.25, *p* = 0.038). In iRBD patients with electrophysiological signs of sensorimotor polyneuropathy, the vibration detection threshold did not significantly differ from those with normal electrophysiological test results. None of the other QST parameters differed significantly between iRBD and HCs (Fig. 2, Tables 2, 3, and Supplementary material). The cold detection threshold on the foot correlated inversely with the NE supine plasma level in iRBD: a higher cold detection threshold was associated with lower NE plasma levels (*r* =  − 0.599, *p* = 0.014).

**Table 2:** QST data of idiopathic REM sleep behaviour disorder patients compared to healthy controls

QST parameter (log and raw data)	Hand		*p* value	Foot		*p* value
	iRBD	HC		iRBD	HC	
Cold detection threshold^log^	**0.44 ± 0.24º**	0.30 ± 0.24	n.s	0.79 ± 0.30*º	0.48 ± 0.24	**0.007**
Warm detection threshold^log^	0.75 ± 0.29	0.60 ± 0.35	n.s	1.02 ± 0.15	0.94 ± 0.19	n.s
Thermal sensory limen^log^	0.97 ± 0.25	0.78 ± 0.31	n.s	1.30 ± 0.18	1.20 ± 0.10	n.s
Cold pain threshold^raw^	7.71 ± 5.66	6.28 ± 2.20	n.s	7.47 ± 4.95	9.71 ± 7.84	n.s
Heat pain threshold^raw^	48.40 ± 1.87	47.58 ± 2.46	n.s	48.76 ± 1.44	48.54 ± 1.58	n.s
Mechanical detection threshold^log^	0.12 ± 0.51	− 0.16 ± 0.58	n.s	0.52 ± 0.46	0.31 ± 0.57	n.s
Mechanical pain threshold^log^	1.82 ± 0.58	1.74 ± 0.45	n.s	1.52 ± 0.49	1.42 ± 0.43	n.s
Mechanical pain sensitivity^log^	0.03 ± 0.56	0.10 ± 0.35	n.s	0.08 ± 0.56	0.23 ± 0.29	n.s
Wind-up ratio^log^	0.29 ± 0.17	0.23 ± 0.17	n.s	0.41 ± 0.21	0.32 ± 0.23	n.s
Vibration detection threshold^raw^	7.11 ± 0.64	7.40 ± 0.73	n.s	5.41 ± 1.79*	6.45 ± 0.93	**0.038**
Pressure pain threshold^log^	2.74 ± 0.11	2.75 ± 0.06	n.s	2.77 ± 0.13	2.76 ± 0.11	n.s

Bold values represent the significant data

Significantly impaired cold and vibration detection thresholds on the foot in iRBD patients compared to HCs. Length-dependent loss of function in cold detection threshold in iRBD patients iRBD (*n* = 17 for all parameters except for thermal testing and PPTfoot *n* = 16) and HC (*n* = 16) on the left hand dorsum and left foot dorsum healthy control (*HC*), idiopathic REM sleep behaviour disorder (*iRBD*), not significant (*n.s.*), quantitative sensory testing (*QST*)

Log transformed data presented as the mean ± SD, *p < 0.05 iRBD vs. HC, *p* < 0.05 Hand vs. Foot in iRBD, *p* values of Fisher’s Least significant difference post hoc test in ANOVA

### Longitudinal autonomic data

Longitudinal data in ten iRBD patients were analysed over a period of 3.64 ± 2.53 years. Missing data included heart rate parameters in two baseline assessments, Valsalva ratio in one baseline and one follow-up assessment; RSA score; Ewing quotient and MoCA in one baseline assessment; and olfaction parameters for the right in two and for the left in one baseline assessment. The maximum systolic BP change (− 24.50 ± 11.70 mmHg at baseline vs. − 42.27 ± 20.01 mmHg at follow-up, *t* =  − 2.72, *p* = 0.024) and maximum diastolic BP change (− 12.79 ± 7.99 mmHg at baseline vs. − 21.60 ± 13.34 mmHg at follow-up, *z* =  − 2.09 *p* = 0.037) were significantly higher at follow-up than at baseline. The heart rate in the upright position was significantly lower at follow-up (70.61 ± 11.71) than at baseline (72.98 ± 10.60 mmHg, *t* =  − 2.93, *p* = 0.019), and the Valsalva ratio was significantly reduced at the follow-up than at baseline (1.46 ± 0.27 BPmax/BPmin at baseline vs. 1.29 ± 0.11 BPmax/BPmin at follow-up, *t* =  − 3.75, *p* = 0.007). None of the remaining parameters changed significantly (Table 1).

## Discussion

This study demonstrates autonomic dysfunction in the form of somatosensory impairment and decreased supine NE levels in iRBD patients compared to those in HCs. Our findings confirm OH as a common autonomic impairment in iRBD patients and reveal progression of OH severity over time. In the present study, our focus was on cardiovascular autonomic function, using sympathoneural neurotransmitter NE values and somatosensory profiles in QST as a non-invasive method for investigating small fibre impairment in iRBD. The demographic and clinical characteristics of the iRBD group are in line with other studies in terms of age, sex, and frequency of autonomic and non-motor symptoms in iRBD [9, 16]. Furthermore, we confirmed previous reports of significantly higher orthostatic BP drops in iRBD patients compared to HCs, as well as hyposmia and increased depressive symptoms as early signs of autonomic and non-motor impairment in iRBD [5, 17–19]. However, it remains unclear if these autonomic impairments in iRBD are more likely due to central or to peripheral dysfunction. Under physiological conditions, plasma levels of sympathetic NE double within five minutes of a postural change, and stabilize BP [14]. In this study, supine NE levels were lower in iRBD patients than in HCs, while the orthostatic NE response was preserved. Various pathologies of NOH in MSA and PD are discussed in the literature. For example, there is a central NE-deficit in MSA patients compared to a peripheral NE-deficit in PD patients [14, 20, 21]. PD patients with NOH demonstrate lower NE supine blood plasma levels in comparison to PD patients without NOH [14]. In iRBD, a peripheral sympathetic dysfunction, such as cardiac sympathetic denervation [9, 22], impaired gut innervation, decreased noradrenergic locus coeruleus innervation, and depletion of neuromelanin-producing cells in locus coeruleus, has been described, implicating a peripheral autonomic impairment in PD patients and underlining the severe autonomic dysfunction in this disease [9]. While in NOH, the NE response to postural change is reduced [14], in our study, the iRBD NE response was maintained. We assume that the lower supine NE blood plasma levels in iRBD patients compared to those in HCs might suggest an early sign of NE-deficit. This hypothesis needs to be validated in longitudinal assessments, investigating possible intra- and inter-individual variability of plasma NE levels, as well as different NE metabolism parameters (e.g., dihydroxyphenylglycol). Competing medication (e.g., serotonin–norepinephrine reuptake inhibitors [23] or beta-adrenergic antagonists [24]), as well as older age [25] might affect norepinephrine metabolism, although all medications, smoking and caffeine intake were paused at least 12 h prior to testing. Since differing entities of our cohort might lead to heterogeneous results, further longitudinal investigations, such as using dopamine transporter or cardiac imaging to discriminate between risks of conversion to PD vs. MSA, may also help to clarify this question. Notably, Knudsen and colleagues [9] recently observed, using multimodal imaging in iRBD, sustained dopaminergic innervation that coincided with impaired autonomic nerve function, noradrenergic dysfunction, and locus coeruleus denervation. In line with this, our study might highlight signs of impairment via NE plasma levels, suggesting a noradrenergic deficiency already present in iRBD with a sustained NE response to postural change. Interestingly, most of the iRBD patients did not experience any orthostatic symptoms, and the initial orthostatic BP drop was often compensated during prolonged standing, which emphasizes the occurrence of subtle autonomic changes prior to clinical manifestation. This also suggests a sustained sympathetic NE response compensating for an initial BP drop. Thus, the significant BP drop in iRBD compared to that in HCs must be caused by other mechanisms (e.g., impaired cardiovagal response). It has been shown that there are neurocirculatory failures, such as an impaired beat-to-beat response to Valsalva manoeuvre in PD patients with OH, MSA or pure autonomic failure [26]. This impairment, over time, was also demonstrated in the present study by means of a significant reduction in the Valsalva ratio and orthostatic heart rate variability response in the longitudinal assessment.

Likewise, as previously described, the QST results in this study confirm the dysfunction of thinly myelinated Aδ-fibres. Unmyelinated C-fibres appear to function physiologically appropriately in iRBD. Compared to a previous study using QST [16], we were able to confirm impaired cold detection on the hand. Additionally, our study showed a length-dependent loss of function for myelinated fibres (Aβ- and Aδ-fibres) with preserved C-fibre performance, as well as significantly impaired cold detection and vibration sense on the foot in iRBD patients compared to those in HCs. Alpha-synuclein aggregates were recently demonstrated in the skin of iRBD patients [10]. In PD patients, these were previously shown in various skin sections (i.e., epidermis [27] and autonomic nerves [28]). Signs of distal sensory loss and small fibre neuropathy [28–30] were also observed. Others have highlighted the presence of sensory impairment in early-stage PD and have reported it to be not a result of therapy [30]. Relating these observations to the present findings in iRBD, our study confirms peripheral somatosensory impairment in this prodromal stage, using QST as a noninvasive technique. Thus, QST might serve as an easy, noninvasive test in diagnosing early loss of function of myelinated fibres in iRBD, confirming the hypothesis of peripheral, rather than central, dysfunction in iRBD. Additionally, when combined with cardiac imaging, QST might serve as a potentially useful biomarker of conversion to α-SYN, and aid in differentiating among different entities with central vs. peripheral impairment.

Limitations of this study include the limited sample size, particularly for the longitudinal data; the lack of additional visual modalities; the lack of central denervation testing; the lack of different NE metabolism parameters (e.g., dihydroxyphenylglycol); the possible confounders in NE metabolism (e.g., age, comorbidities, medications); and the possible intra- and inter-individual variability of plasma NE levels.

As there are recent theories on different subtypes in PD [31], iRBD is proposed to be part of a severely affected phenotype [32]. The disease is associated with faster progression of non-motor and motor symptoms [32] and early sympathetic cardiac denervation [9]. iRBD in PD might be associated with a higher disease burden, higher risk of mortality, and poor prognosis [33]. Zitser et al. described an association with severe autonomic dysfunction and a greater risk of phenoconversion and severe progression [34].

Overall, this study confirms early peripheral autonomic dysfunction in the form of somatosensory impairment and decreased NE plasma levels, which is in line with the peripheral-to-central α-SYN spreading hypothesis. The findings support the concept of a peripheral origin and suggest autonomic testing as a feasible method to find predictive markers of conversion to α-SYN and future-directed potential protective therapies. This is important in light of a severe phenotype and poor prognosis. Further longitudinal investigations of QST, autonomic assessment, and NE levels may clarify the progression and severity of autonomic dysfunction in iRBD.

## Supplementary Information

Below is the link to the electronic supplementary material.

Supplementary file1 (DOCX 23 KB)

## Data Availability

None.
