# Supplementary material for: Quantitative sensory testing and norepinephrine levels in REM sleep behaviour disorder – a clue to early peripheral autonomic and sensory dysfunction?
Source: J Neurol. 2021 Jun 25;269(2):923–32. doi: 10.1007/s00415-021-10675-7 (PMC8782803; doi:10.1007/s00415-021-10675-7)
Supplement: Supplementary file 1 — Supplementary file1 (DOCX 23 KB) [file 415_2021_10675_MOESM1_ESM.docx]

**Table 3 supplementary material**

Heading Somatosensory profile of idiopathic REM sleep behaviour disorder patients compared to healthy controls

| ANOVA iRBD vs. HC | | | | |  |  |
| --- | --- | --- | --- | --- | --- | --- |
|  | (1) Main factor group (“between factor”) | | (2) Main factor region (hand vs. foot; “within factor”) | | (1) + (2) Interaction | |
|  | F Value | p Value | F Value | p Value | F Value | p Value |
| **Cold detection threshold** | 8.97 | **0.005** | 2.36 | n.s. | 2.36 | n.s. |
| Warm detection threshold | 3.36 | n.s. | 0 | n.s. | 0 | n.s. |
| Thermal sensory limen | 5.41 | **0.027** | 0.45 | n.s. | 0.45 | n.s. |
| Cold pain threshold | 0.21 | n.s. | 1.79 | n.s. | 1.79 | n.s. |
| Heat pain threshold | 0.72 | n.s. | 0.31 | n.s. | 0.31 | n.s. |
| Mechanical detection threshold | 2.68 | n.s. | 0.09 | n.s. | 0.09 | n.s. |
| Mechanical pain threshold | 0.46 | n.s. | 0.01 | n.s. | 0.01 | n.s. |
| Mechanical pain sensitivity | 0.58 | n.s. | 0.95 | n.s. | 0.95 | n.s. |
| Wind-up ratio | 1.88 | n.s. | 0.00 | n.s. | 0 | n.s. |
| **Vibration detection threshold** | 4.33 | **0.046** | 1.91 | n.s. | 1.91 | n.s. |
| Pressure pain threshold | 0.07 | n.s. | 0.57 | n.s. | 0.57 | n.s. |

Caption Somatosensory profile indicating loss of function for myelinated fibres in iRBD patients. Data was calculated based on z-scores independent of the body regions

iRBD (n=17 for all parameters except for thermal testing and PPTfoot n=16) and

HCs (n=16)

healthy control (HC), idiopathic REM sleep behaviour disorder (iRBD), not significant (n.s.), quantitative sensory testing (QST)
